# Facilitating and supporting the engagement of patients, families and caregivers in research: the “Ottawa model” for patient engagement in research

**DOI:** 10.1186/s40900-022-00350-0

**Published:** 2022-06-07

**Authors:** Shelley Vanderhout, Stuart Nicholls, Zarah Monfaredi, Claudia Hampel, Lynn Ashdown, Maxime Bilodeau, Susan Rich, Beverley Shea, Dean Fergusson

**Affiliations:** 1 Clinical Epidemiology Program, Ottawa Hospital Research Institute, 501 Smyth Road, Box 201B, Ottawa, ON K1H 8L6 Canada; 2 School of Epidemiology and Public Health, University of Ottawa, 600 Peter Morand Crescent, Room 101, Ottawa, ON K1G 5Z3 Canada; 3 Faculty of Health Sciences, Simon Fraser University, Blusson Hall, Room 11300, 8888 University Drive, Burnaby, BC V5A 1S6 Canada; 4 Patient Relations, The Ottawa Hospital, 1053 Carling Avenue, Box 133, Ottawa, ON K1Y 4E9 Canada; 5 Patient Partner Expert, Faculty of Medicine, University of Ottawa, 451 Smyth Rd #2044, Ottawa, ON K1H 8M5 Canada; 6 Patient Partner, The Ottawa Hospital, 1053 Carling Ave, Box 133, Ottawa, ON K1Y 4E9 Canada; 7 Patient Partner Expert, The Ottawa Hospital, 1053 Carling Ave, Box 133, Ottawa, ON K1Y 4E9 Canada; 8 Faculty of Medicine, University of Ottawa, Roger Guindon Hall, 451 Smyth Rd #2044, Ottawa, ON K1H 8M5 Canada

**Keywords:** Patient engagement, Framework, Capacity building, Model

## Abstract

**Background:**

Patient engagement is increasingly being recognized as a critical component of health research; however, institutional models for building infrastructure and capacity for patient engagement in research are limited. There is an opportunity to create reproducible and scalable models of patient engagement in research and share best and promising practices.

**Main body:**

In this article, we describe the development and features of the framework for the Ottawa Patient Engagement in Research Model at The Ottawa Hospital (TOH) and the Ottawa Hospital Research Institute (OHRI). Key components of the model include: a Patient and Family Engagement Program at TOH, which recruits, educates, and supports patients, families and caregivers to engage in clinical care, governance, and research; the Ottawa Methods Centre within the OHRI, which leads methodological research and provides support to investigators for patient engagement and patient-oriented research at TOH; and the Office of Patient Engagement in Research Activities, also within the OHRI, which facilitates collaborations between patients, researchers, clinicians and other stakeholders. Early success of this model can be attributed to aligned institutional priorities between TOH, OHRI and patients, the establishment of a patient engagement policy, ongoing education and support provided to patient partners and researchers, and innovative recruitment, tracking and evaluation procedures. Ongoing challenges and next steps include promoting diversity among patient partners, implementing an equitable compensation policy, engaging patients across a variety of roles and research areas, and developing resources to expand and sustain this program.

**Conclusion:**

This model represents a unique effort of patients, clinicians, researchers, and policymakers across disciplines and institutions to produce a harmonized strategy and infrastructure for meaningful collaboration with patients and families in health research, and capacity building in patient-oriented research.

**Supplementary Information:**

The online version contains supplementary material available at 10.1186/s40900-022-00350-0.

## Plain English summary

Involving patients and families as partners in planning, conducting, and sharing results of health research, referred to as patient engagement, is becoming more common and recognized as important part of the research process. However, guidelines and examples of how to do this well are limited. In this article, we describe the development and features of the Ottawa Patient Engagement in Research Model at The Ottawa Hospital (TOH) and the Ottawa Hospital Research Institute (OHRI). Key pieces of the model include: a Patient and Family Engagement Program, which recruits, educates, and supports patients and families to engage in clinical care, decision making, and research; the Ottawa Methods Centre, which leads studies to understand the best methods to conduct research, and provides support to researchers for patient engagement; and the Office of Patient Engagement in Research Activities, which connects patients, researchers, clinicians, and other stakeholders. Early success of this model may be a result of shared priorities between TOH, OHRI and patients, the creation of a patient engagement policy, ongoing support provided to patients, family members and researchers, and creative methods for recruitment, tracking and evaluation procedures. Ongoing challenges and next steps include promoting diversity among patient partners, setting up a fair and transparent policy for compensating patient partners, and engaging patients across a variety of roles and research areas.

This model represents a unique effort of patients, clinicians, researchers, and policymakers across disciplines and institutions to produce one strategy for meaningful teamwork with patients and families in health research.

**Supplementary Information:**

The online version contains supplementary material available at 10.1186/s40900-022-00350-0.

## Background

In health research, there has been an increasing recognition of the importance of patient, family, and caregiver engagement, which can include partnering with patients in priority setting, developing research methods and outcomes [1–7], co-designing and conducting aspects of research, and communicating findings collaboratively [8]. The term “patient” is used to capture a range of individuals who can be involved in a person’s health care: people with health conditions, their caregivers, and others with relevant lived experience [9]. Patient partnerships are intended to result in patient-oriented research, which involves and engages patients across the research process and focuses on questions and outcomes relevant to them [4]. Systematic reviews have identified benefits of patient engagement in research such as increased acceptability and accessibility of evidence-based care options and greater relevance of health research topics; however, challenges such as tokenism (including a patient voice, but largely ignoring it [10]) and a lack of diversity in patient partners are common [11–13].

Efforts to formalize patient engagement in health care, research and governance originated in the UK, with the development of INVOLVE (now part of the National Institute for Health Research) in 1996 [14]. Subsequently, other national entities have been established to support patient engagement in research, including the US Patient Centered Outcomes Research Institute (PCORI) in 2010 [15] and the federal Canadian Institutes for Health Research (CIHR) Strategy for Patient Oriented Research (SPOR) in 2013, which encourages Canadian researchers and health care decision-makers to engage patients, families, and caregivers as collaborators in all stages of health research, as well as health care governance [4]. The Ontario SPOR Support Unit (OSSU) [16] established in 2015 through CIHR and Ontario government funding, had a specific mandate to support the development of patient engagement strategies across Ontario research communities. In Ontario, the Patients First Act (2016) [17] mandated that Patient and Family Advisory Committees (PFACs) be established at the ministry and hospital levels to focus on advising on issues germane to clinical care experience.

The same cannot be said for research. Despite international models of engagement and organizations such as INVOLVE and PCORI, there is a dearth of information regarding *institutional* models and approaches to building infrastructure and capacity for patient engagement in research [18, 19]. As Canada continues to grow patient-oriented research programs, there is an opportunity to create reproducible and scalable models of patient engagement in research and share practices.

## Main text

### Objective

The aim of this paper is to support the process of sharing experiences and describe the joint development and features of the Ottawa Patient Engagement in Research Model at The Ottawa Hospital (TOH) and Ottawa Hospital Research Institute (OHRI). Consistent with the CIHR definition, patient engagement refers not simply to the engagement of patients but is used as an “overarching term inclusive of individuals with personal experience of a health issue and informal caregivers, including family and friends” [4]. The roles and responsibilities of the Ottawa Methods Centre (OMC), the Office of Patient Engagement in Research Activities (OPERA), and The Ottawa Hospital Patient and Family Engagement Program (PFEP) are described in building our institutional policy, infrastructure, and support. A summary of early successes and opportunities for improvement is also provided.

### Setting

The Ottawa Hospital is a large academic tertiary care centre comprised of three hospital campuses in Ottawa, Canada, with a total of 1200 beds serving a population of over 1.3 million in communities across Eastern Ontario and Nunavut. The Ottawa Hospital Research Institute is the research arm of TOH and one of its strategic research priorities is practice-changing research [20], which places emphasis on connecting research outcomes directly to clinical care. By orchestrating a concerted effort between researchers, clinical staff, hospital administration, and patients, families, and caregivers, the Ottawa Hospital Research Institute has championed patient engagement in research. A glossary of acronyms used in this paper can be found in Box 1.

**Box 1:** Glossary

CIHR	Canadian Institutes of Health Research
eNOI	Electronic notice of intent
IAP2	International Association of Public Participation
IMPACT	Innovative, Measurable, Patient-oriented, Appropriate, Collaborative
OHRI	Ottawa Hospital Research Institute
OHSN-REB	Ottawa Health Science Network Research Ethics Board
OMC	Ottawa Methods Centre
OPERA	Office for Patient Engagement in Research Activity
OSSU	Ontario Strategy for Patient-Oriented Research Support Unit
PCORI	Patient Centred Outcomes Research Institute
PFAC	Patient and Family Advisory Council
PFEP	Patient and Family Engagement Program
SPOR	Strategy for Patient-Oriented Research
TOH	The Ottawa Hospital

### Institutional drivers and vision for the model

**Fig. 1:**
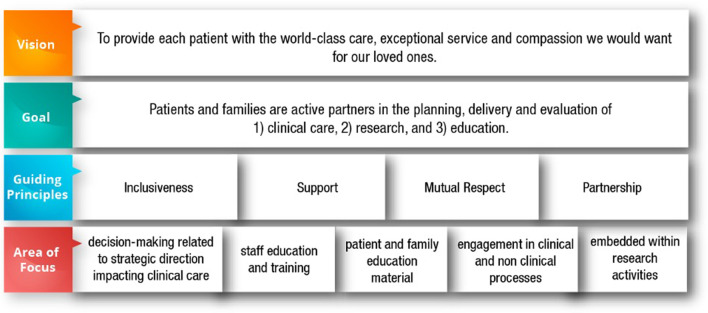
The Ottawa Hospital Patient Engagement Framework. Based on the Canadian Institutes for Health Research Strategy for Patient Oriented Research Capacity Development Framework [27]

**Fig. 2:**
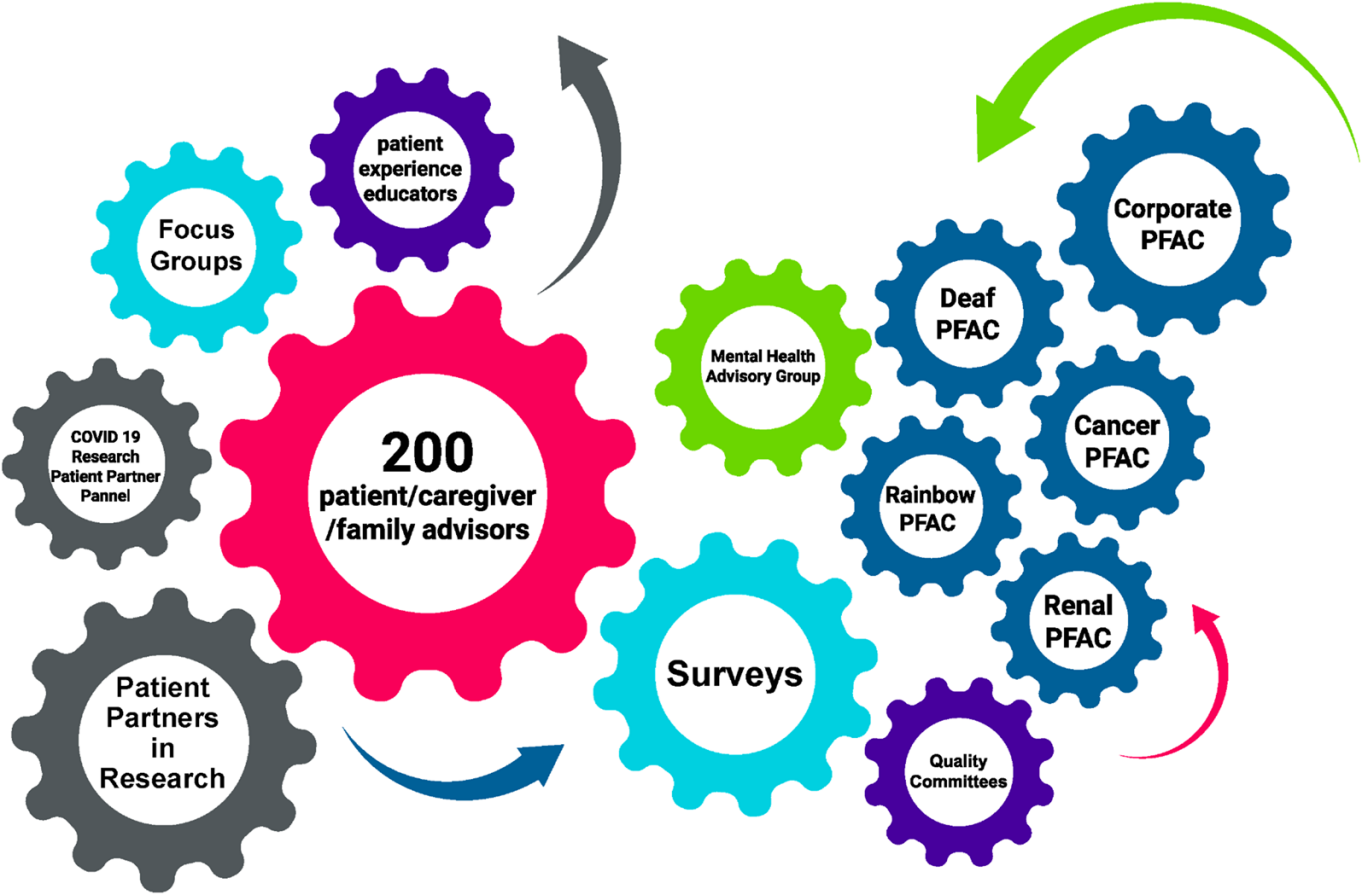
Patient and Family Advisory Council (PFAC) structure for The Ottawa Hospital

**Fig. 3:**
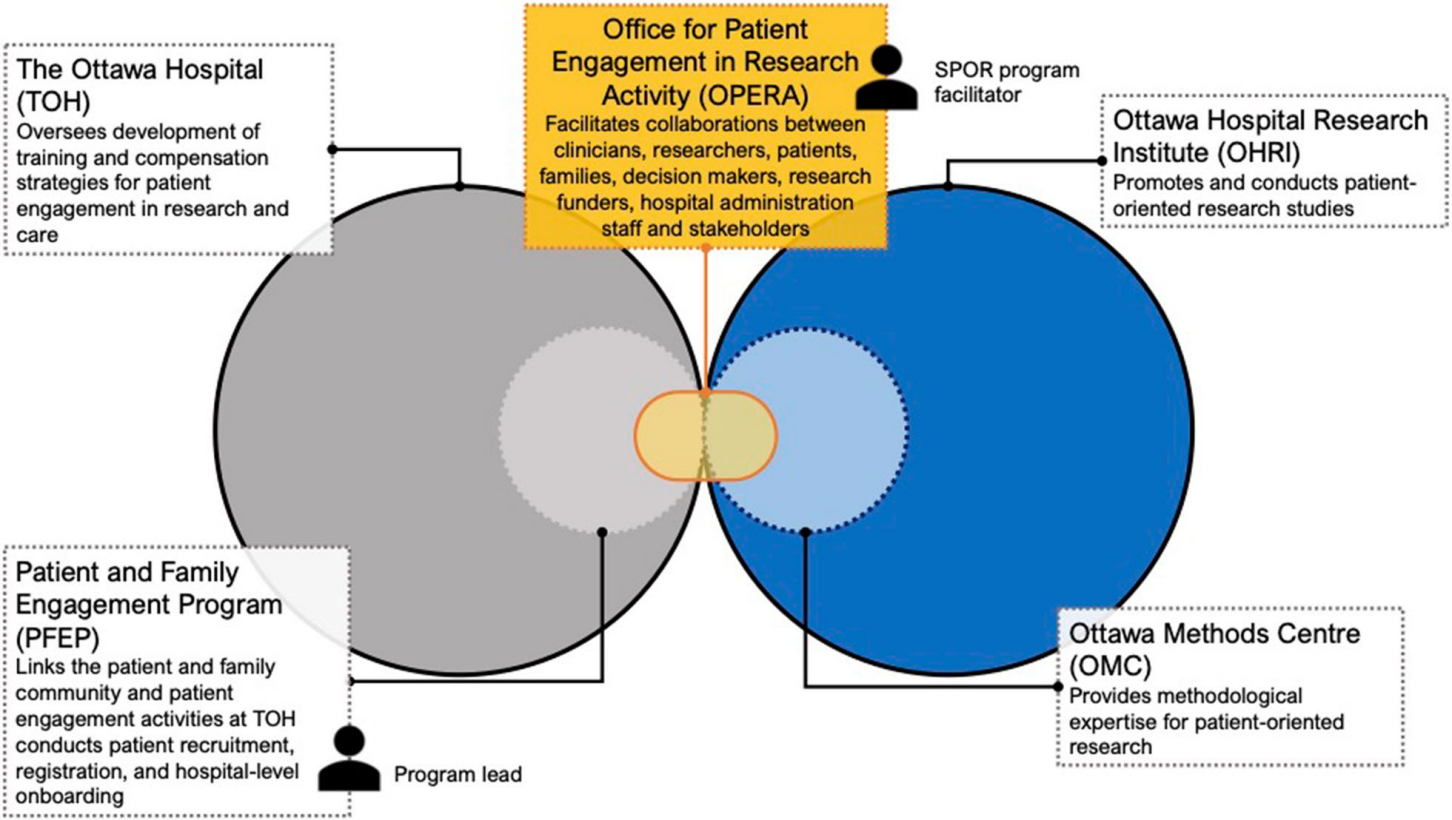
Pillars of support for The Ottawa Patient Engagement in Research Model. *SPOR* Strategy for Patient Oriented Research

Creation of the Ottawa Model for Patient Engagement in Research was initiated by a need to develop expertise and infrastructure in alignment with the launch of CIHR’s SPOR in 2013 [4]. In 2014, the first Patient and Family Advisory Committee (PFAC) was created at TOH and has subsequently grown into the Patient and Family Engagement Program (PFEP), comprised of multiple PFACs and joint staff-patient/caregiver committees and projects. Need for support for patient engagement at the hospital level was catalyzed by both the Ontario government’s Patients First Act (2016) [17] and the OSSU (2015) [16] mandate to implement patient engagement across health care and research institutes in Ontario. With the provincial infrastructure and mandate in place, a harmonized vision and goal across both TOH and the OHRI was developed based on the SPOR guiding principles [21] (Fig. 1). Importantly, while TOH and OHRI exist as two formal entities, the shared vision reflects a commitment to mutual learning and collaboration with respect to patient engagement where research is integrated within the broader vision. This vision has been supported by the development of the Office for Patient Engagement in Research Activities, a formalized structure of collaboration between the PFEP at TOH and the OMC within the OHRI (Table 1, Figs. 2, 3).

**Table 1:** Supports and services for researchers and patients at The Ottawa Hospital and Ottawa Hospital Research Institute

	For researchers—office of patient engagement in research activities (OPERA)	For patients—patient and family advisory program (PFEP)
Consultations	Protocol guidance, methods support	Guidance on attending meetings with research teams, and advising as a patient partner
Education	Foundations of patient-oriented research, educational seminars led by local experts in the field	Foundations of patient-oriented research, hospital and research orientation
Hospital resource navigation	Identification of contacts for internal patient committees, human resources, and the Ottawa Health Science Network Research Ethics Board	Supports for navigating compensation, human resources, and the Ottawa Health Science Network Research Ethics Board

### Collaborative infrastructure and personnel

With a mandate to promote a culture of patient and family centered care at TOH, the PFEP is the overarching program responsible for the development and support for PFACs and individual patient partners. A unique feature of the PFEP is that it supports researchers, patient partners, and multiple committees which focus on specific areas of research or care, such as weight management, cancer, and renal health (Fig. 2). While the mandate of the PFEP is primarily to support patient engagement in care and governance, its resources and activities overlap and support patient engagement in research at TOH and OHRI. Responsibilities of the PFEP include the recruitment of patient partners, registration, introductory hospital-level onboarding, and orientation. In addition to patient partners actively engaged as members of the various PFACs, the PFEP also supports an active roster of patient partners who may contribute as individuals to specific activities within the hospital or research institute and who are matched on a project-by-project basis. A dedicated Patient and Family Engagement Lead serves to coordinate the PFEP at TOH and provides support to patient partners engaged in care, governance, and research. As part of this role, the Patient and Family Engagement Lead, works within OPERA to facilitate the matching and support of patient partners within research. As such, the PFEP and Patient and Family Engagement Lead serve as the essential links between patients (and families) and research activities.

The Ottawa Methods Centre (OMC) is a core facility within OHRI and is also a member of the province-wide network of centres that form the OSSU [16]. The OMC is comprised of scientists and staff who provide research expertise and support to researchers affiliated with the OHRI and TOH, and provides methodological expertise to investigators pursuing patient-oriented research. The OMC offers a range of services including research design and methodology support, data management and big data analytics, statistical consultation and health economics, knowledge translation and evidence implementation support. It is also an OSSU-designated methods support unit for patient-oriented research and knowledge translation for the province. To facilitate and support patient-oriented research, OPERA is operationally based within OMC and includes a dedicated individual, the SPOR Program Facilitator (SGN), who provides consultations with researchers as well as methods training. The SPOR Program Facilitator role has four core components; first, they work with researchers and research teams directly to advise on how to engage patients and to share resources (for example, tools and templates that can be used in grants). Second, they provide education and outreach to the research and patient community. This outreach ranges from aware- ness-raising about patient-oriented research and patient engagement through to targeted education on specific approaches to research and engagement. Third, they work with the PFEP program lead, to facilitate the matching of patient partners from the TOH Patient and Family Engagement Program with research teams. Finally, the SPOR Program Facilitator, together with OMC scientists and OPERA staff, engage in research to advance the methods of patient engagement and patient-oriented research.

### Features of The Ottawa Patient Engagement in Research Model

In addition to the development of the vision, relationships, and collaborative infrastructure to support patient engagement in research at TOH and the OHRI, several steps have been taken to support patients, families and caregivers as well as researchers. These include: adopting a patient engagement in research framework (above); developing a formal patient engagement policy at OHRI; establishing approaches to engagement; building expertise in patient engagement methods; developing processes for matching patients, families, and caregivers with researchers; leading education initiatives for both researchers and patient partners; creating resources for patient-oriented research; and developing and implementing systems to track and evaluate patient-oriented undertaken through TOH and the OHRI.

### Policy

A Patient Engagement Policy was implemented in 2020 at the OHRI which encourages all levels of staff, from senior management to trainees, to implement patient engagement in research activities. The policy, available to all staff and trainees, provides information on the patient engagement framework, guiding principles, an overview of services provided through OPERA, as well as recommendations regarding compensation for patient partners in research [22, 23]. The policy serves to delineate the roles and responsibilities of OHRI senior management, PFEP, OPERA, and OMC as they relate to supporting patient engagement in research.

### Approaches to engagement

Patient engagement in research at the OHRI typically occurs at the project level, with patients, families and caregivers engaged in specific projects with defined goals and for a finite period. Patient-researcher partnerships may extend across projects, but this is largely dependent on research funding for a specific project. In most cases, researchers seek input or collaboration as they develop research questions, objectives, and study protocols, select outcome measures, recruit participants, and interpret and communicate results [8]. Consequently, the structure for patient engagement in research tends to follow what Greenhalgh et al. have described as a study-focused framework [24]. Examples of research supported by OPERA are provided in Table 2. Beyond patient engagement at the project level, we anticipate growth for patient partners in research institutional governance, priority setting, and development of patient-centred outcomes. Indeed, patient advisors from the PFEP were recently consulted as part of the review of strategic planning and priorities for the OHRI.

**Table 2:** Examples of research supported by the Office of Patient Engagement in Research Activities (OPERA)

Study/program	Description	Outputs
Operating Room Black Box Research Program	During a 12-month implementation period, the team conducted 23 stakeholder engagement activities with over 200 participants	Fifteen patients and 17 perioperative clinicians were interviewed, which identified key themes to include in an information campaign run as part of the implementation process. Two patient partners were engaged and advised on communications as well as grant and protocol development [38, 39]
Getting better outcomes with chimeric antigen receptor T-cell therapy (GO-CART) program	Chimeric antigen receptor (CAR) T-cell therapy is a class of personalized treatment for blood cancers, where some of a patient’s own T-cells are removed, genetically modified in a laboratory to specifically target and kill cancer cells, then re-administered to the patient. The team led an early phase clinical trial to assess the safety and effectiveness of a novel CAR-T treatment for resistant blood cancer	Given evidence that patient partners may improve the development and conduct of clinical trials, the team applied a novel integrated knowledge translation (iKT) approach to engage patients (from inception) in the development of the CAR-T cell therapy phase I/II clinical trial protocol to ensure that clinical trial processes and resources were aligned with patient needs [26]
PREDICT app	The team developed the Personalized Risk Evaluation and Decision Making in Preoperative Clinical Assessment (PREDICT) app, a tablet application that was calibrated to local data, followed best practices for risk communication, and leveraged surgery patients’ ability to provide and receive their own health information	Patients gained knowledge of personalized risk of adverse events and reported improved satisfaction after elective surgeries [40]

### Undertaking research into the methods of patient engagement

In order to support researchers and patient partners with the most current advice and methods support, OPERA leads and engages with methodological research in the field of patient-oriented research and patient engagement. This has included a systematic review of patient engagement roles within clinical trials [25], case studies of patient engagement within an early-phase cancer treatment research called GO-CART [26], working with other researchers to develop engagement evaluation frameworks [27] as well as internally-led work to explore the effect of patient engagement on research funded through OSSU. Examples of the latter include interviews with IMPACT (Innovative, Measurable, Patient-oriented, Appropriate, Collaborative) grant awardees regarding the influence that patient engagement had on their studies [28], and survey research to explore the perspectives of journal editors regarding patient co-authors [29].

### Identification and matching of patient partners

As indicated above, a key component of the work undertaken by OPERA is matching researchers with patients, families, and caregivers from the PFEP. In order to support this role, OPERA has developed several approaches that allow existing clinical patient partners to be matched with research projects, and also allow patients, families, or caregivers to proactively indicate an interest in partnering with research and to identify clinical areas of interest.

In the first instance, researchers may submit an online request form through the OMC which captures key details about the project including the project title, project stage, funding status, deadline, and a brief overview of the support being sought. Submission of the form activates a matching process, where the SPOR Program Facilitator liaises with the researcher and the Patient and Family Engagement Lead, who can then identify potential patient partners from the pool of partners who have signed up within the PFEP. While this requires a matching process and data curation, by drawing from a larger pool of advisors and having centralized information, the Patient and Family Engagement Lead can manage requests and workload for patient advisors while also ensuring a range of perspectives can be brought to bear on research. These patient partners are then contacted by the Patient and Family Engagement Lead with a description of the project, and if they express an interest, they are introduced to the research team. Alternatively, a request for a partner may come directly to the PFEP with an indication that the project involves research engagement. In this case the Patient and Family Engagement Lead would coordinate with the SPOR Program Facilitator and, as above, arrange a consultation.

Epic MyChart (epic.com) is the institutional electronic medical record system at TOH, which includes a secure online patient portal that gives patients access to their medical and personal health information. MyChart allows patients to access a range of medical information including after-visit summaries, upcoming appointments, and to review test results. Patients, families, and caregivers who receive care at TOH can use their MyChart electronic health record to express a general interest in participating in research and give permission for researchers to contact them. Patients further can select clinical areas relevant to their experiences to facilitate matching them with related research projects. Patients are carefully matched to activities suited to their skills, interests, and experiences to maximize impact and positive experience. This is intended to reduce potential for tokenism by ensuring that patient partner experiences are brought to research where they are most relevant, and increase the probability that patient partners will feel empowered to contribute their perspectives. Importantly, the program actively encourages early discussion of roles and responsibilities and developing Terms of Reference (or similar). Recently developed systems also create automatic distribution of evaluation surveys when projects reach their end dates meaning that patient and researcher perspectives are sought, with a view to addressing any challenges identified. The active and ongoing engagement of patients and researcher-patient teams indicates that the approach has been successful. If patients are selected by researchers, the Program Lead from the PFEP contacts them about being an advisor at TOH and arranges for them to learn about the role and participate in an orientation workshop. Future MyChart developments will include the creation of surveys distributed through MyChart to capture more granular data on individual experiences, as well as to provide information about ongoing patient-oriented research at TOH and OHRI and opportunities to engage with researchers.

### Education

A key element to the work undertaken by OPERA is ongoing education, both for the patient partners and researchers engaged in patient-oriented research, which was collaboratively developed by the Patient and Family Engagement Lead and a patient partner. Education activities include webinars on discrete topics, such as Open Science, as well as Storytelling workshops that support patient partners to learn how to communicate their healthcare experiences in a way that will impact research and medical education [30]. Onboarding of patient partners is facilitated by an online volunteer management system which collates patient partner contact information and provides oversight of patient partner matching and assignment. Patients are supported by the Patient and Family Engagement Lead and attend orientation sessions (based on evidence when available) to learn what to expect throughout their role, how to share their personal experiences effectively, and provided resources to support their participation such as navigating sensitive or challenging topics, confidentiality, and reporting issues. Through this process, patients are encouraged to connect with peer partners in effort to broaden and strengthen the patient engagement community at TOH. As with other activities, patient and staff education initiatives actively include patients, families, and caregivers; for example, patients have participated in developing educational websites, creating a patient declaration of values, and presenting as patient teachers at institution-wide rounds. Patient partners who have worked with study teams are also involved in delivering lectures or seminars about those projects and OPERA works with the teams to support the inclusion of patient partners.

For researchers, education may involve self-directed learning and utilization of resources produced by OPERA, attending lectures and seminars [16], as well as individualized training on patient-oriented research methods and patient engagement offered and supported by the SPOR Program Facilitator. The OPERA team has been active in the development of a patient-oriented research curriculum [31], supported by the CIHR SPOR [21]. In addition, the OPERA team also actively presents at local, provincial, national, and international conferences and have written popular media pieces with patient partners [32, 33].

### Tools and resources

To promote accessible resource sharing across research disciplines and clinical areas, an employee-facing SharePoint site has been developed for all TOH and OHRI staff looking to engage patients in their work. This site, accessible internally and updated on an ongoing basis, provides plain language dictionaries, relevant literature, evaluation tools, templates developed at TOH for patient engagement materials, and a checklist to ensure research teams are ready to work alongside patient partners (see Additional File 1: *Checklist for engaging patients in research*). This site is also the point of entry for the ‘Request a Partner’ form where staff can submit requests for patient partners as indicated above.

### Tracking

Monitoring patient engagement across research is paramount to understanding current strengths and identifying areas for improvement. OPERA has worked with institutional partners to integrate capturing patient engagement data within several research processes.

First, data regarding the involvement of patient partners is captured within the electronic Notice of Intent (eNOI) process. The eNOI is part of the institutional process established by the OHRI and through which researchers notify the institution of their intention to submit for grant funds. OPERA worked with the OHRI to include in the eNOI an item that asks, “Are patients included as partners in research (examples include helping define the research question or inform study design)?”. If the researcher indicates Yes, then an email alert is sent to the SPOR Program Facilitator who contacts the researcher to see if a consultation is required and, if so, how they can help. Going forward, this system will facilitate a better understanding of the number and proportion of studies engaging patients from study conception and will allow for analyses such as proportion of studies receiving funding and whether patient engagement at this stage enhances funding success rates.

Second, and following the implementation of the patient engagement policy at the OHRI, the Ottawa Health Science Network Research Ethics Board added fields about patient engagement to its research ethics application forms. This includes a general question as to whether patients are engaged, consistent with the question asked as part of the eNOI process. If the researcher indicates that patients have or will be engaged, then further questions are asked exploring the aspects of the study where patients, families, or caregivers have been or will be engaged. This information allows OPERA to track patient engagement in research following grant success or in unfunded studies and across clinical domains and time. In addition, by tracking these data, the OPERA team can identify potential areas for training or support; for example, if patients are not being engaged in specific research or clinical areas.

### Evaluation

In partnership with TOH, a Patient and Family Evaluation Working Group at OHRI was established to support an evaluation plan for the Patient and Family Engagement Program in research and care, which is based on priorities identified by patient partners to improve patient experiences. This working group develops tools to evaluate the overall program and patient partner experiences, analyzes evaluation data collected via surveys and emails, and supports employee training and orientation based on feedback. Working with the OMC and within OPERA, these tools have been expanded and adopted to evaluate patient engagement in research, both from the researcher and patient partner perspectives. Concerns and compliments are filed, reviewed, and followed up by the Patient and Family Engagement Lead. Going forward, as new tools and frameworks for patient engagement in research are developed, these evaluation forms will be reviewed and revised by the OPERA team and patient partners to ensure that meaningful and useful results translate into improvements in the engagement of patients in research.

### Strengths of the Ottawa Model for Patient Engagement in Research

Key successes and challenges of the model, and next steps are shown in Table 3. One of the key strengths of the Ottawa Model for Patient Engagement in Research is that both entities are in strategic alignment, including strong support from senior management at both institutions. The OPERA has created a dedicated centralized institutional hub for patient engagement in research activities, and facilitates interdisciplinary communication, efficient implementation and tracking of patient engagement, clear messaging about the approach to patient engagement in research, and opportunities for patients to participate in both research and hospital-focused partnerships. Leadership and coordination provided by patient engagement specialists at TOH and OHRI are another strength of this patient engagement model as engaging patients remains novel to many clinicians and researchers. Centralized support and resource management also ensures patient engagement is well conducted. Importantly, the patient engagement strategy at TOH and OHRI runs across the spectrum of basic to clinical to applied research [26]. This model also includes each of the features considered essential to building capacity for patient engagement in health care settings by patients, clinicians and hospital administrators: resources, training, organizational commitment, and staff support [34].

**Table 3:** Key successes, challenges, and next steps

Successes	Challenges	Next steps
• Strategic alignment and support from senior management at TOH and OHRI • Coordinated resources, training, organizational commitment, and staff support for patient engagement in research • Patient partners engaged in a spectrum of basic to clinical research	• Limited resources for staff to match, educate and support researchers and patient partners, and for ongoing information technology and evaluation • Equitable, harmonized compensation	• Understand current patient partner demographics and how representation of the TOH patient community can be improved • Equip researchers to work with diverse groups in culturally appropriate ways • Evaluate patient and researcher experiences

### Challenges with the model to date

Being a new program that developed organically, the model has experienced challenges. At present, the Patient and Family Engagement Lead is the only full-time employee managing a pool of almost 200 patients who wish to be engaged in clinical areas or research across both TOH and OHRI. A strength of the present program has been the ability to maintain contact and interactions with patient partners, but expanding patient engagement in research without increased staff support may negatively impact the program. Moreover, the manual matching of patient advisors with research teams is labor and time intensive. Mechanisms and strategies that may facilitate this approach are currently being reviewed. Similarly, the SPOR Program Facilitator is a single position with a broad range of responsibilities. While the dedicated position is a major asset, and perhaps a rarity, it remains a soft-funded position dependent on large scale project investment in entities such as OSSU. Sustained resources are needed to support various aspects of patient engagement such as information technology, evaluation, and capacity building. Ongoing work to capture and evaluate data on the impact of patient engagement will be essential to demonstrating benefit and offering the evidence to create the necessary stable and properly resourced platform upon which to build cross-institutional patient engagement.

We have an ongoing need for solutions to compensating to patient partners. While compensation is explicitly acknowledged in the patient engagement policy at the OHRI, it raises challenges for cross institutional harmonization, such as administrative barriers and lack of plain-language resources to help patients understand possible implications of compensation (for example, on other financial supports they may receive). While researchers are supported to budget for and provide compensation for patient partners’ time and reimbursement for expenses such as transportation, parking and meals, this patient engagement model does not recommend any single mechanism of compensation due to the varied nature of patient engagement obligations. Moreover, while researchers may be able to support compensation, the PFEP has no budget to provide payment or honorarium to patient partners. This creates an inequity whereby patient partners are not compensated for their input on clinical activities, but may be compensated for input into research. The complexity of this issue has resulted in inconsistent practices, which need to be resolved moving forward. There is a need for overarching best practice guidelines for compensating patient partners in care and research, including practical guidance on methods of compensation and issues to be aware of.

### Diversity

Ensuring that all patients are represented by patient partners, and barriers to participation are minimized so all interested patient partners can participate, are critical components of incorporating all relevant perspectives in patient engagement [35, 36]. The activities of the PFEP, OPERA, and OMC are in alignment with TOH’s broader diversity strategy as well as OHRI’s emerging diversity work through its Equity, Diversity, & Inclusion (EDI) council. The established recruitment approach through MyChart aims to reach all patients who receive care at TOH, which is intended to improve the diversity of patient partners, but carries limitations such as requiring computer literacy and English or French comprehension. Training provided to researchers includes specific information about overcoming difficulties to engagement and connecting with underrepresented groups, with the aim of intentionally creating an accessible and comfortable space for patients.

Capacity building in this space is underway. For example, TOH has developed a Rainbow PFAC that has a cross cutting remit to inform issues pertaining to sex and gender and is collaborating with the SPOR program facilitator to identify priorities for research teams or inform practices around data collection for sex and gender. ‘Fairness is Excellence’ equity diversity and inclusion training, is available to researchers through OSSU, and Ownership Control Access and Possession (OCAP) training, pertaining to Indigenous health data, is mandatory where applicable. Next steps include developing an overarching compensation policy for patient partners at the OHRI, which will address financial barriers to engagement and support equitable inclusion of patient partners, and building patient partner representation into the OHRI EDI council.

### Next steps

Our model certainly has room for growth and improvement. As the identification of partners through MyChart is in early stages, there will be opportunities to better optimize this tool once evaluation of its reach and performance is possible, such as creating targeted efforts to reach underrepresented patient populations and expand PFEP membership to individuals who represent missing demographics. Concurrently, researchers may need support to acquire knowledge on working with diverse groups in culturally appropriate ways. Oversight will be needed to ensure that patient engagement activities are in alignment with TOH’s broader diversity strategy as well as OHRI’s emerging diversity work through its Equity, Diversity, & Inclusion council. Data from MyChart and research protocol information from Research Ethics Board applications about patient engagement uptake will be analyzed to develop further improvements, including understanding who is currently engaged and how representation of the diverse TOH patient community can be improved. In addition to high-level evaluation of patient engagement in research, implementing routine feedback from researchers to patient partners and vice versa may promote continuity of engagement and result in stronger relationships. The education provided as part of the orientation process will be updated regularly as additional evidence emerges, and all patient partners will be supported to have agency to choose their engagement across care and research, according to their skills and interests. Several basic and translational research groups at TOH have engaged patients to date, but this is an area with potential for growth in patient engagement. Types of patient engagement identified at TOH and the OHRI are typically consulting, informing, and involving patients, but ideally more patients would have collaborative and empowerment roles, consistent with the International Association of Public Participation (IAP2) spectrum [37]. Providing patient partners with an orientation to research in addition to the current orientation program may also help to equip patients for roles in research. Finally, as the OHRI patient engagement policy was recently implemented in 2020, it will be revised and updated as needed with the help of the OSSU.

## Conclusion

The Ottawa Model for Patient Engagement in Research represents a broad collaboration between researchers, clinicians, methodologists, patient engagement experts, research ethics board members, patients and families. Development of this model has involved numerous stakeholders and resulted in the creation of various supporting groups of experts. This patient engagement model is established as a recognized infrastructure across clinical and research disciplines, and may provide a transferrable framework for other institutions who are engaging patients and families in health research.

## Supplementary Information

**Additional file 1.** Checklist for engaging patients in research.

## Data Availability

Not applicable.
