# Supplementary material for: Facilitating and supporting the engagement of patients, families and caregivers in research: the “Ottawa model” for patient engagement in research
Source: Res Involv Engagem. 2022 Jun 7;8:25. doi: 10.1186/s40900-022-00350-0 (PMC9172149; doi:10.1186/s40900-022-00350-0)
Supplement: Supplementary file 1 — Additional file 1. Checklist for engaging patients in research. [file 40900_2022_350_MOESM1_ESM.docx]

**Additional File 1: Checklist for engaging patients in research**

This document is for Principal Investigators to use as a guide for initial onboarding of patient partners in a research study

*For the intents and purposes of this document, “patient partner” will be used to refer to any person with experience of an illness or disease, including family and friend caregivers.*

1. The patient partner(s) has/have been informed about the research study and type of research that we are conducting

*This may include relevant background information about how the health system works, what research is, how research informs health care decisions, research methodology to be used in the study, etc.*

- Yes
- No

1. I have all the contact information that I need for the patient partner(s) and the contact information of each team member has been shared with them.

- Yes
- No
- In progress

1. I have identified a person on the research team to act as a Patient Engagement Lead

*The Patient Engagement Lead will be responsible for ensuring the patient partner(s) on the team are continuously engaged and that the team remains accountable to the patient engagement goals and responsibilities (as detailed by the Terms of Reference- see #6 below). They will also act as a point person for the patient partner(s) to contact as needed.*

- Yes. I, the project PI, will be the Patient Engagement Lead
- Yes. I have identified another member of the research team to be the Patient Engagement Lead.
- No

1. The Patient Engagement Lead has contacted the patient partner(s) ahead of scheduling the first team meeting

*It is important to establish a connection with the patient partner(s) as early as possible. Before scheduling the first team meeting, you should have a basic understanding of each patient partner’s availability and any accessibility requirements they may have.*

- Yes
- No
- In progress

1. The patient partner(s) have been introduced to the team and the team has been introduced to the patient partner(s)

- Yes
- No
- We plan for the first agenda item at our first team meeting to be an introduction.

1. I have a draft Terms of Reference (**to be finalized with patient partner input**)

- Yes
- No
- In progress
- I need help (Template from NIHR INVOLVE: [Terms of Reference](file:///C:\Users\snicholls\Consultations\TERMS%20OF%20REFERENCE.docx))

1. All of my privacy, confidentiality, and risk management concerns have been addressed and discussed with my research team (including patient partner(s))

*All advisors must be registered with the TOH Patient Engagement Program and must sign confidentiality agreements with TOH*

*Information from their applications/interviews is confidential and cannot be shared without their consent.*

- Yes
- No
- Not applicable

1. The communications tools that will be used during this project have been explained to the patient partner(s)

*Examples: teleconference, web-based meetings like GoTo Meeting, online calendar*

- Yes
- No
- Not applicable
